# Dataset regarding the mechanical characterization of sedimentary rocks derived from Svalbard for possible use in local road constructions

**DOI:** 10.1016/j.dib.2021.106735

**Published:** 2021-01-09

**Authors:** Diego Maria Barbieri, Jean-Gabriel Dorval, Baowen Lou, Hao Chen, Benan Shu, Fusong Wang, Inge Hoff

**Affiliations:** a Department of Civil and Environmental Engineering. Norwegian University of Science and Technology, Høgskoleringen 7A, Trondheim, 7491 Trøndelag, Norway; b Department of Civil Engineering, Technical University of Denmark, Brovej Building 118, Kongens Lyngby, 2800 Hovedstaden, Denmark; c Foshan Transportation Science and Technology Co. Ltd, Wuhan University of Technology, Kuiqi second road 18, Foshan, 528000, Guangdong, China; d State Key Laboratory of Silicate Materials for Architectures, Wuhan University of Technology. Luoshi road 122, Wuhan 430070, Hubei, China

**Keywords:** Pavement unbound, Road construction materials, Los Angeles test, Micro-deval test, Repeated load triaxial test, Longyearbyen, Svalbard

## Abstract

The dataset deals with the mechanical characterization of sedimentary rocks collected along the banks of Longyear river in proximity of Longyearbyen (Svalbard) at the junction of Bolterdalen and Adventdalen valleys. As the rocks represent possible local construction materials that can be employed in the new road infrastructures located in the Svalbard archipelago, three types of laboratory investigations were performed for mechanical characterization: Los Angeles tests, micro-Deval tests and repeated load triaxial tests. The grading curve of the material characterized with the repeated load triaxial tests corresponded to a typical one commonly adopted in Norway for road base layer (0–31.5 mm). The dataset offers a thorough overview of the mechanical properties relevant for road constructions and the dataset can be useful to both contractors and transportation agencies operating in the Svalbard archipelago.

## Specifications Table

**Table:** 

Subject	Civil and Structural Engineering
Specific subject area	Road pavement engineering, Material testing
Type of data	Table Image Graph Figure
How data were acquired	The data were collected by performing the following laboratory tests: Los Angeles (LA) test, micro-Deval (MDE) test, Repeated Load Triaxial Test (RLTT)
Data format	Raw analysed
Parameters for data collection	Los Angeles (LA) tests, micro-Deval (MDE) tests and Repeated Load Triaxial Tests (RLTTs) were performed according to codes EN 1097-2, EN 1097-1 and EN 13286-7, respectively. Specimens with two water contents (*w* = 2% and *w* = 7%) were investigated by means of RLTTs
Description of data collection	The analysed materials were rocks derived from the banks of Longyear river in proximity of Longyearbyen (Svalbard) at the junction of Bolterdalen and Adventdalen valleys. Los Angeles (LA) tests, micro-Deval (MDE) tests and Repeated Load Triaxial Tests (RLTTs) were performed in the laboratory
Data source location	The rock materials were collected in Svalbard, the coordinates of the specific location are 78°10′34.0″N 15°58′11.2″E. The testing campaign was performed at the Department of Civil and Environmental Engineering, Norwegian University of Science and Technology (NTNU), Høgskoleringen 7A, Trondheim 7491, Norway
Data accessibility	Dataset is uploaded on Mendeley Data Repository name: Mechanical characterization of sedimentary rocks derived from Svalbard for possible use in local road constructions Data identification number: DOI: 10.17632/mtn347g3gp.1 Direct URL to data: https://data.mendeley.com/datasets/mtn347g3gp/1

## Value of the Data

• The data are related to the main mechanical properties that need to be ascertained when using rocks as construction aggregates in road infrastructures. The characterized materials could be employed for local purposes in the Svalbard archipelago.
• The data can be useful for both contractors and transportation agencies operating in Svalbard when considering the suitability of the local rocks for road constructions. The dataset can also be beneficial to researchers operating in the sector.
• The data can be effectively employed to characterize a rock material type available in the Svalbard archipelago and assess its feasible use as aggregates for local road constructions.
• The possibility to make use of local materials in civil infrastructures located in the Svalbard archipelago is relevant as it can reduce the importation of aggregates from continental Norway.

## 1 Data Description

The dataset refers to the mechanical characterization of sedimentary rocks collected in Svalbard; the information contained in the dataset can be useful to assess the performance of the rocks considering their possible local use as road construction materials. The accomplished investigation campaign encompassed three laboratory investigations: Los Angeles (LA) test, micro-Deval (MDE) test and Repeated Load Triaxial Test (RLTT). The dataset is publicly available (https://data.mendeley.com/datasets/mtn347g3gp/1).

**Fig. 1:**
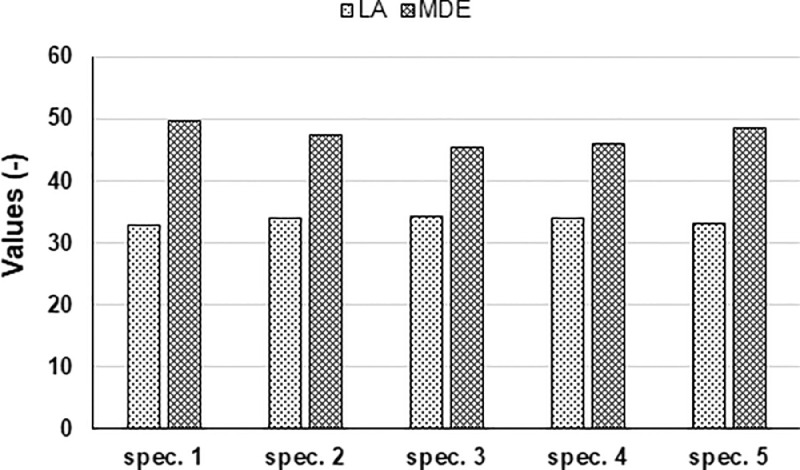
Los Angeles (LA) and micro-Deval (MDE) values of each tested specimen.

The spreadsheet “Los Angeles and micro-Deval results.xlsx” reports on LA and MDE. As displayed in Fig. 1, five parallel samples were tested and the average values were 33.6 and 47.3, respectively. These outcomes can be compared to the thresholds defined by pavement design guidelines, i.e. Norwegian guidelines [1].

**Fig. 2:**
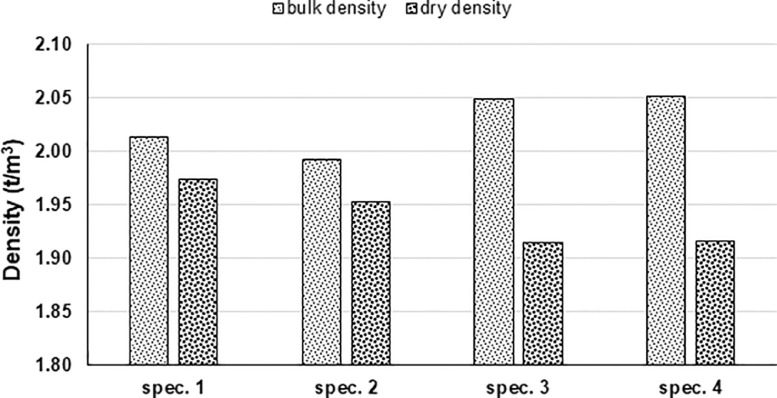
Bulk density and dry density of each specimen investigated with RLTTs.

Four specimens were investigated with the RLTTs, two of them (specimens 1 and 2) had water content *w* = 2% and two of them (specimens 3 and 4) had water content 7%. Fig. 2 displays the bulk density and the dry density for each tested sample [2].

The data corresponding to RLTTs are reported in “Repeated Load Triaxial Tests results - specimen 01 - water content 2%”, “Repeated Load Triaxial Tests results - specimen 02 - water content 2%”, “Repeated Load Triaxial Tests results - specimen 03 - water content 7%” and “Repeated Load Triaxial Tests results - specimen 04 - water content 7%” files, all the four spreadsheets are structured in the same fashion. Each spreadsheet is composed by five sheets corresponding to each RLTT loading sequence. Columns A, B, C, D, E report the step number (six steps in total), time *t* since the sequence started, temperature *T* (namely room temperature), deviatoric pulse number and frequency *f* (fixed to 10 Hz), respectively. Columns F and G display the dynamic and the static part of the deviatoric stress *σ_d_* exerted vertically by the hydraulic jack, the former one (*σ_d,dyn_*) varies as described in the next section, the latter one (*σ_d,st_*) is always approximately equal to 5 kPa to guarantee a contact between the jack and the metal end-platen. Columns H and I display the dynamic and the static part of the triaxial stress *σ_t_* exerted by the pressurized water surrounding the specimen, the former one (*σ_t,dyn_*) is always approximately equal to 0 kPa and the latter one (*σ_t,st_*) varies for each sequence as described in the next section. The axial deformations measured by the three vertical Linear Variable Displacement Transformers (LVDTs) are reported in Columns J, L, N (elastic components *ε_a,el,_*_1_, *ε_a,el,_*_2_, *ε_a,el,3_*) and Columns K, M, O (plastic components *ε_a,pl,_*_1_, *ε_a,pl,_*_2_, *ε_a,pl,3_*). The radial deformations measured by the three horizontal LVDTs are reported in Columns P, R, T (elastic components *ε_r,el,_*_1_, *ε_r,el,_*_2_, *ε_r,el,3_*) and Columns Q, S, U (plastic components *ε_r,pl,_*_1_, *ε_r,pl,_*_2_, *ε_r,pl,3_*).

**Fig. 3:**
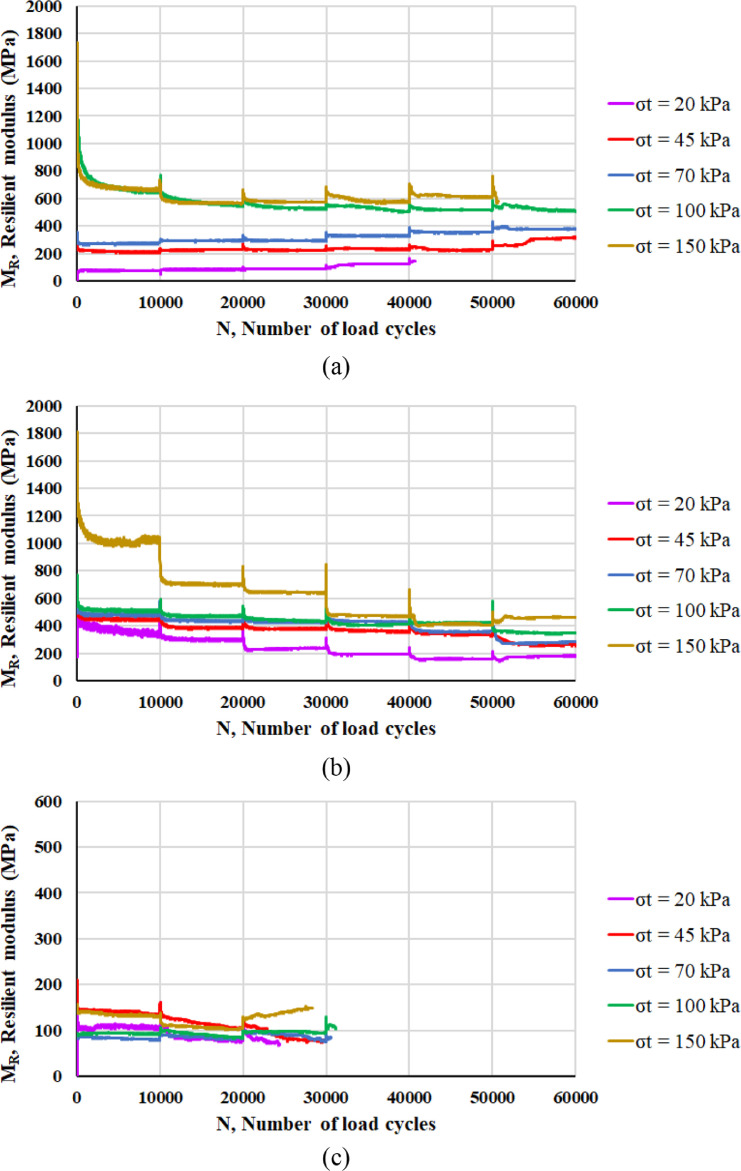

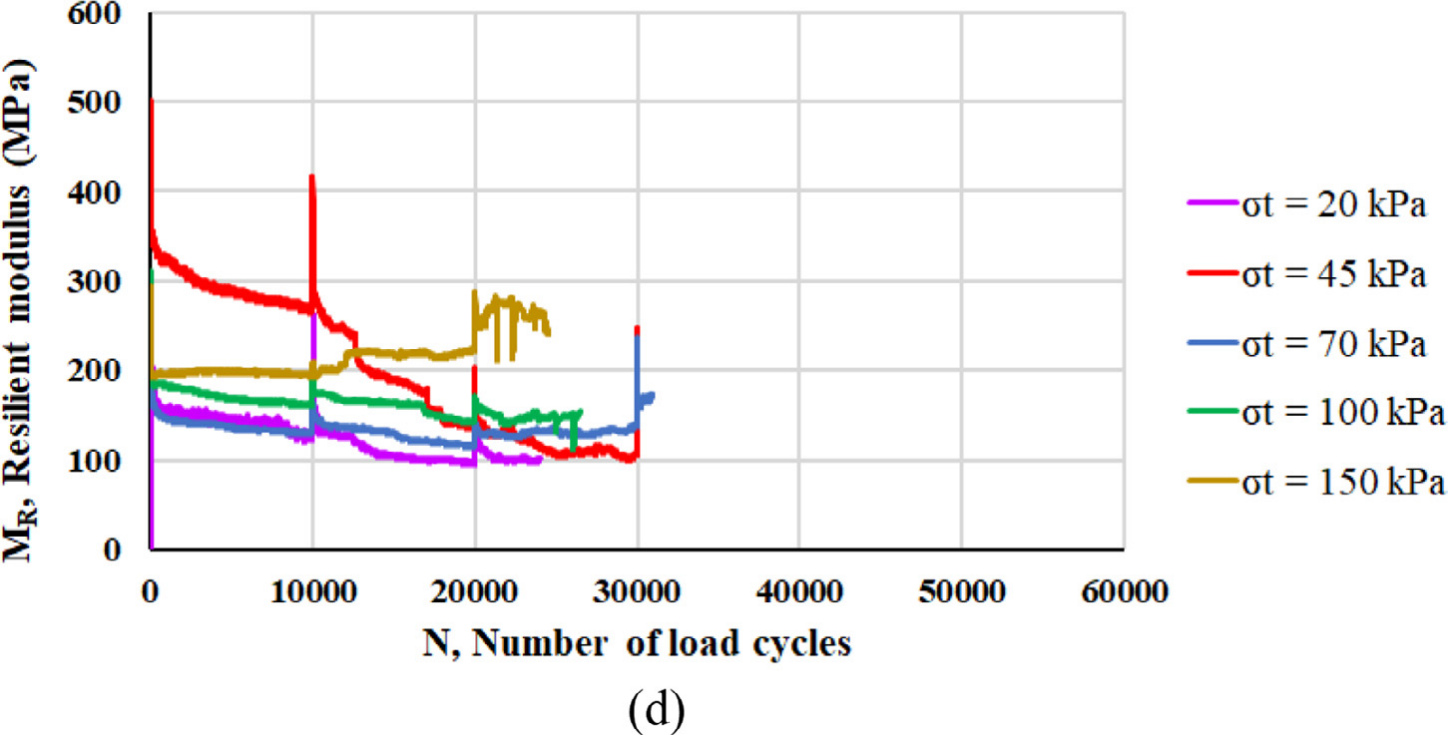
Resilient modulus *M_R_* and number of load cycles *N* for specimen 1 (a), specimen 2 (b), specimen 3 (c) and specimen 4 (d).

The two main properties that are assessed are the resilient modulus *M_R_* and the resistance against permanent deformation. Following the definition of resilient modulus *M_R_* detailed in the next section, Fig. 3 reports the values of *M_R_* corresponding to each RLTT sequence (*σ_t_* = 20 kPa, 45 kPa, 70 kPa, 100 kPa, 150 kPa) for specimen 1 (Fig. 3a), specimen 2 (Fig. 3b), specimen 3 (Fig. 3c) and specimen 4 (Fig. 3d) according to the number of load cycles *N*.

**Fig. 4:**
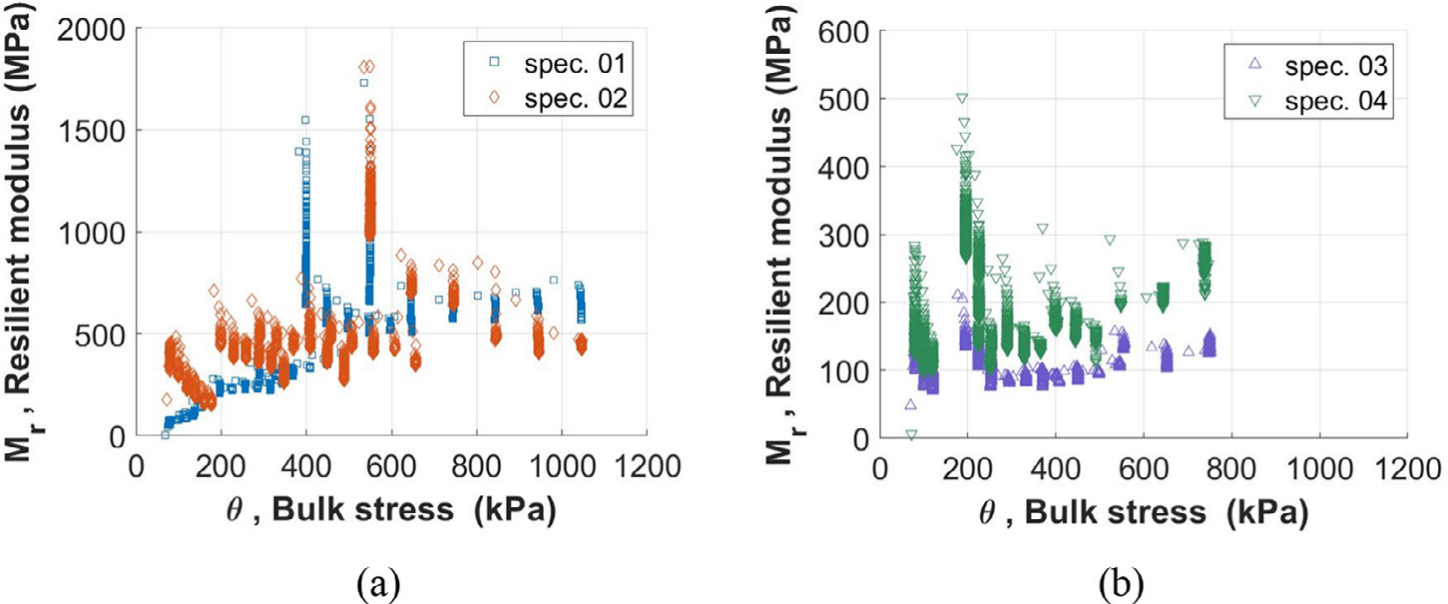
Resilient modulus *M_R_* and bulk stress *θ* for specimens 1 and 2 with *w* = 2% (a) and for specimens 3 and 4 with *w* = 7% (a).

Furthermore, as reported in Fig. 4, all the experimental data can be also plotted in a two-dimensional space displaying bulk stress *θ* and resilient modulus *M_R_* along *X*-axis and *Y*-axis, respectively, for *w* = 2% (Fig. 4a) and *w* = 7% (Fig. 4b).

**Fig. 5:**
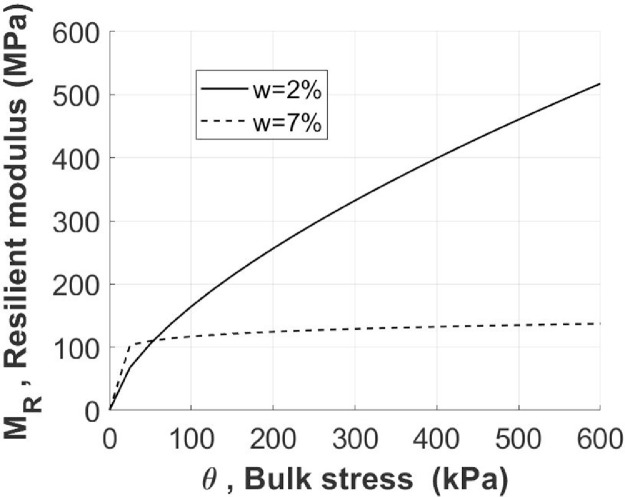
Resilient modulus *M_R_* and bulk stress *θ* for specimens with *w* = 2% and *w* = 7% according to Hicks and Monismith model.

Considering this two-dimensional space, the overall trend can be efficiently calculated adopting the Hicks & Monismith regression model as illustrated in Fig. 5 and described in the next section. Table 1 reports the values of the regression parameters *k_1_, k_2_*.

**Table 1:** Regression parameters *k_1_, k_2_*.

	k_1_	k_2_
*w* = 2%	1646.2	0.64
*w* = 7%	1169.7	0.09

**Fig. 6:**
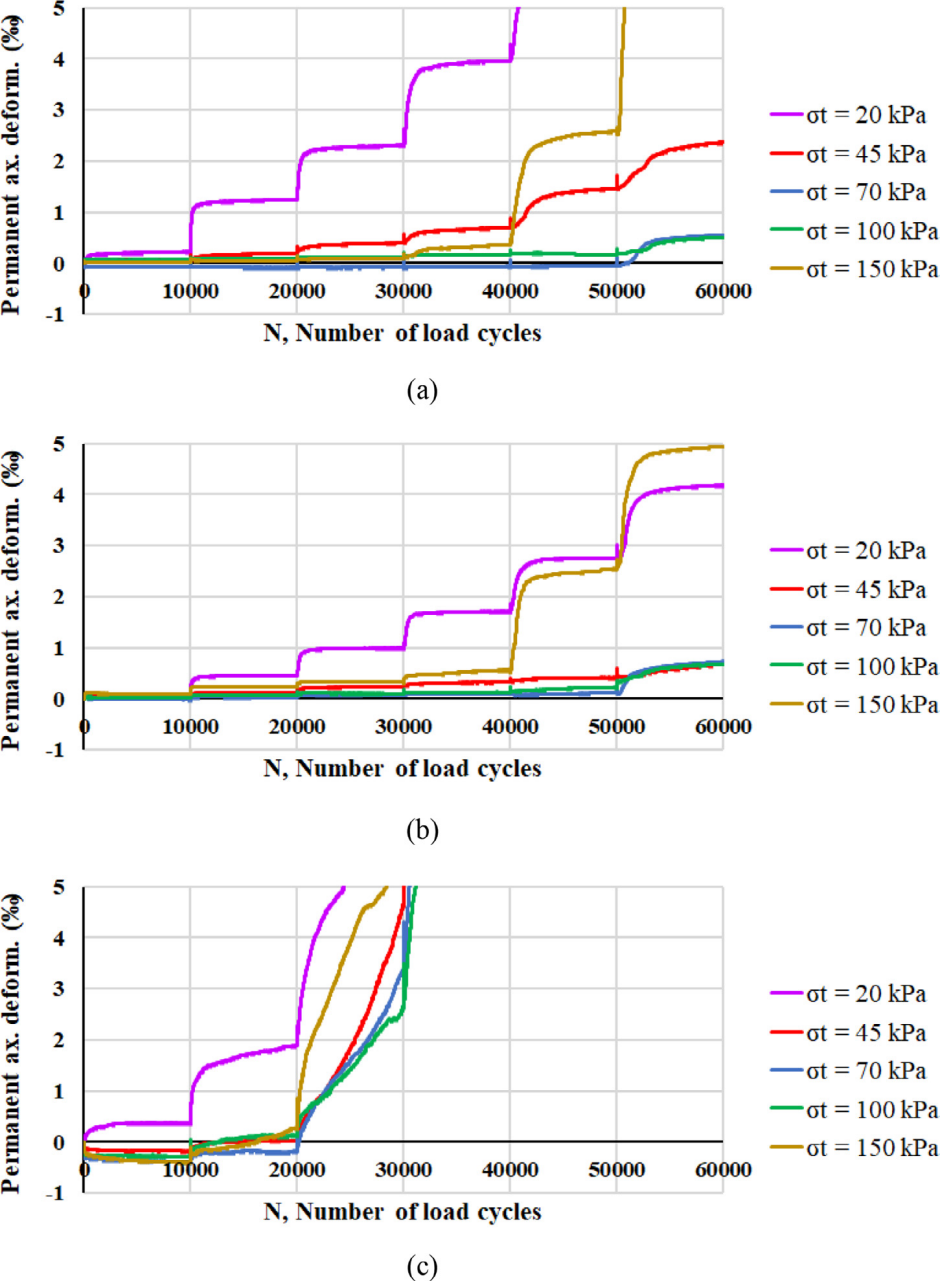

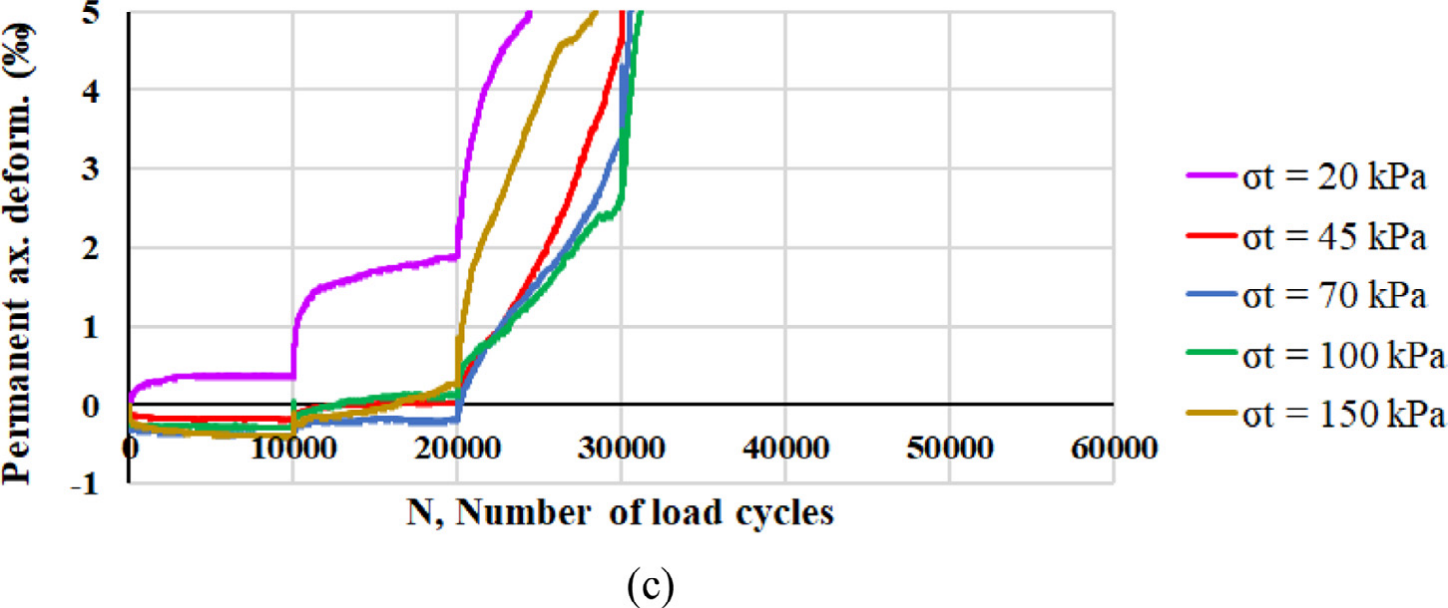
Axial plastic deformation *ε_a,pl_* and number of load cycles *N* for specimen 1 (a), specimen 2 (b), specimen 3 (c) and specimen 4 (d).

Considering the development of axial plastic deformations, Fig. 6 reports the values of *ε_a,pl_* corresponding to each RLTT sequence (*σ_t_* = 20 kPa, 45 kPa, 70 kPa, 100 kPa, 150 kPa) for specimen 1 (Fig. 6a), specimen 2 (Fig. 6b), specimen 3 (Fig. 6c) and specimen 4 (Fig. 6d) according to the number of load cycles *N*.

**Fig. 7:**
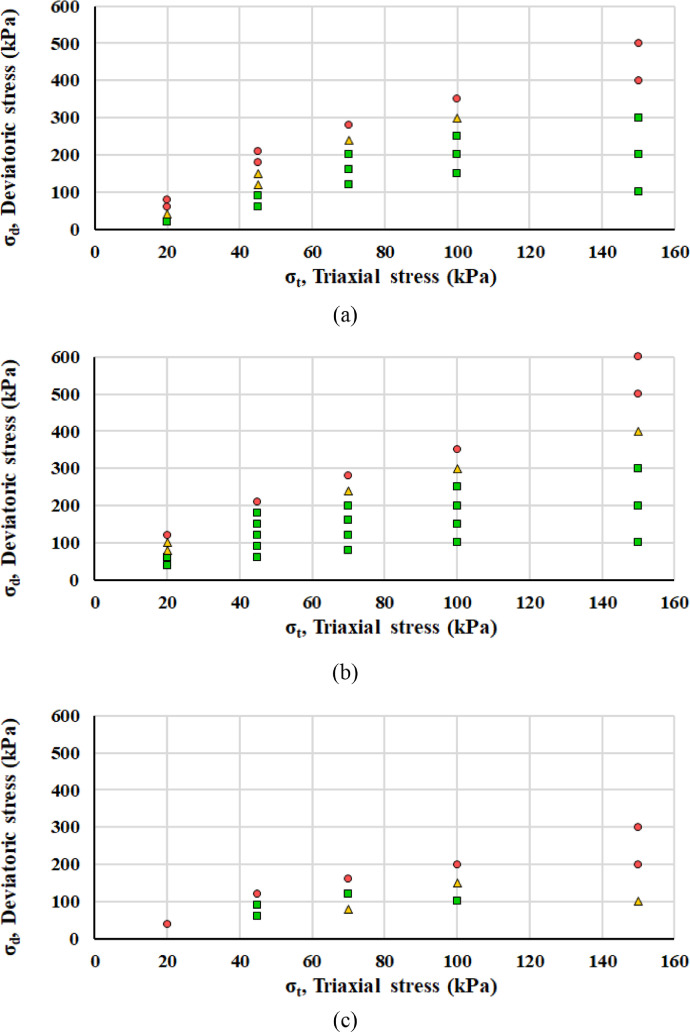

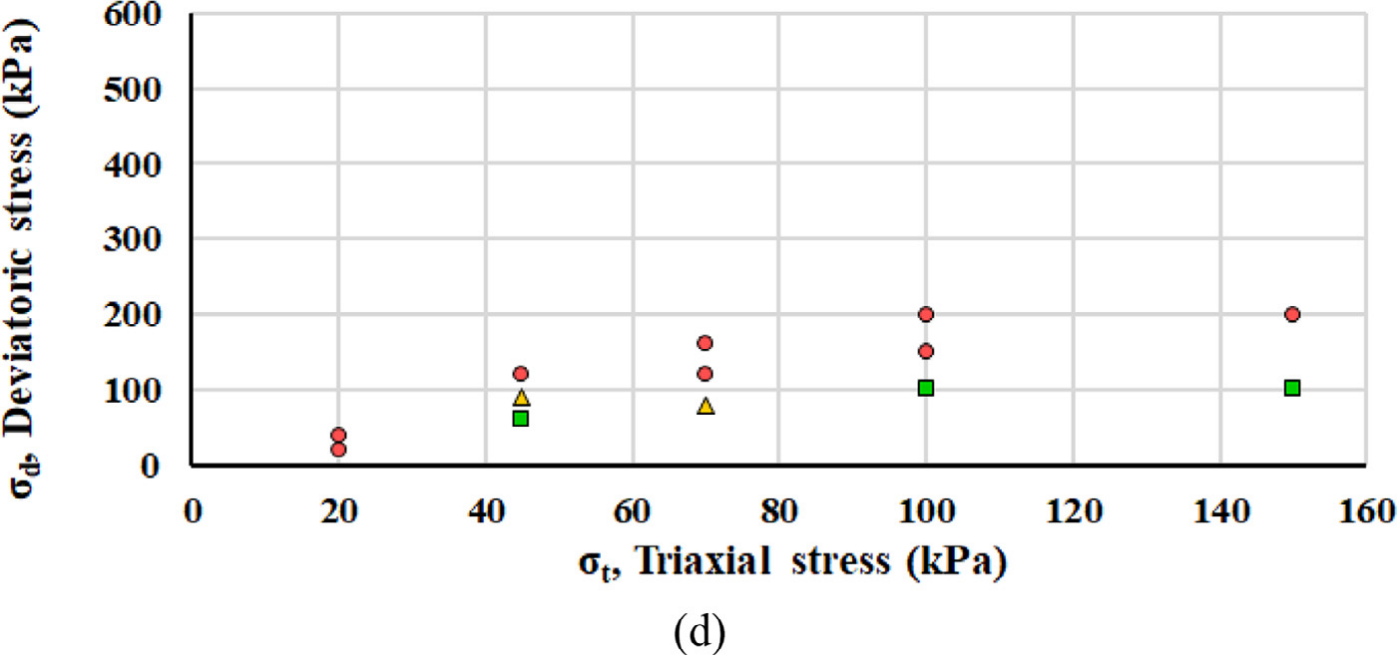
Classification of each RLTT loading step according to the Coulomb approach for specimen 1 (a), specimen 2 (b), specimen 3 (c) and specimen 4 (d).

**Fig. 8:**
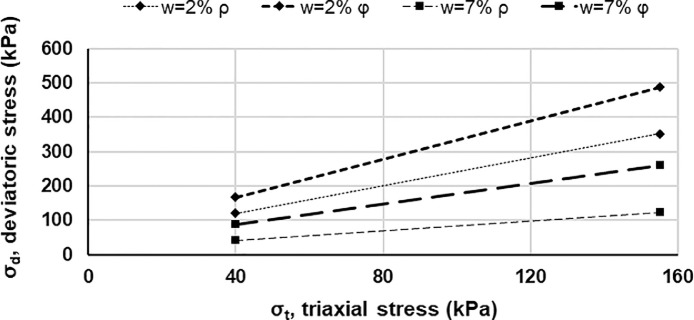
Mobilized angle of friction *ρ* and angle of friction at incremental failure *φ* for specimens with *w* = 2% and *w* = 7% according to the Coulomb approach.

Interpreting the axial plastic deformations according to the Coulomb approach described in the next section, each load step belongs to elastic, elasto-plastic or failure range: as reported in Fig. 7, green squares, yellow triangles and red circles symbolize elastic, elasto-plastic or failure range, respectively. Fig. 8 displays the mobilized angle of friction *ρ* and the angle of friction at incremental failure *φ*.

## 2 Experimental Design, Materials and Methods

**Fig. 9:**
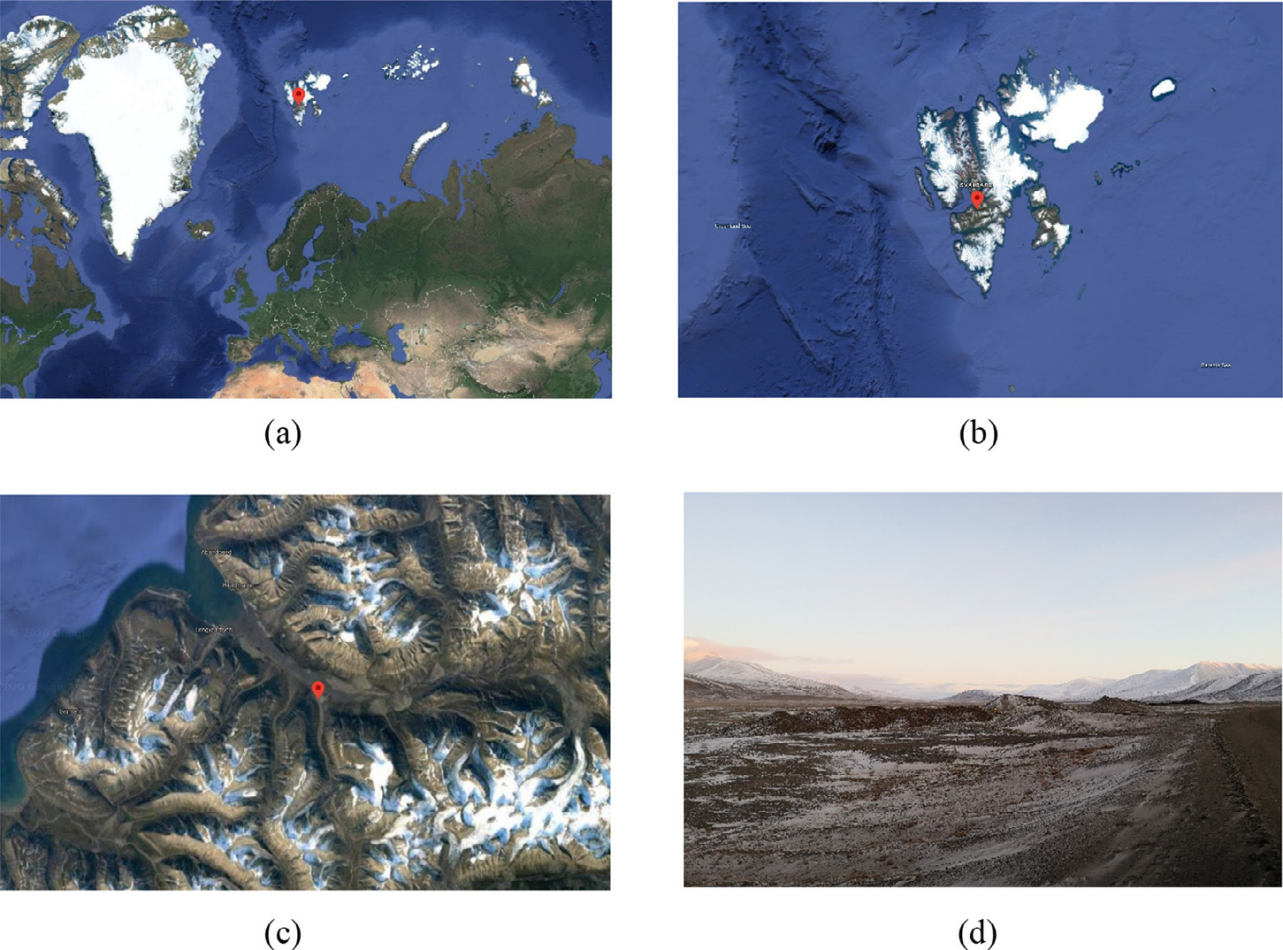
Geographical location where the tested rock materials were collected; Fig. 9a, 9b and 9c are obtained from Google Maps [3].

The tested rock materials originated from the banks of Longyear river in proximity of Longyearbyen (Svalbard) and were collected at a stockpile near the junction of Bolterdalen and Adventdalen valleys, the coordinates of the specific position reported in Fig. 9 are 78°10′34.0″N 15°58′11.2″E, Fig. 9a, 9b and 9c are obtained from Google Maps [3]. The rock materials available in the Svalbard archipelago mostly have sedimentary origin [4].

**Fig. 10:**
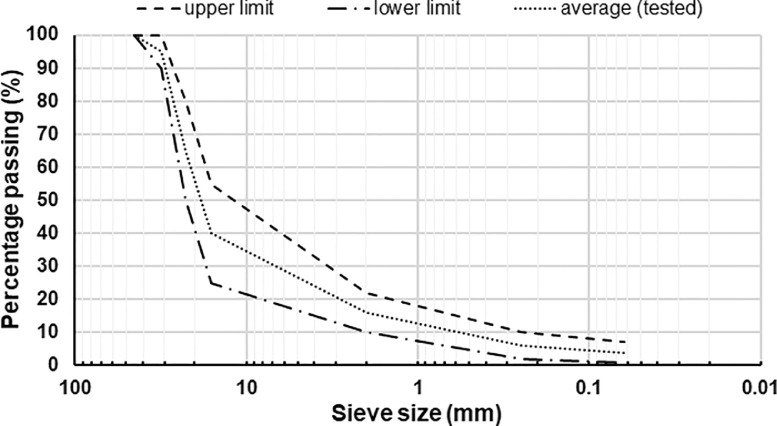
Particle size gradation tested with RLTTs [1].

**Fig. 11:**
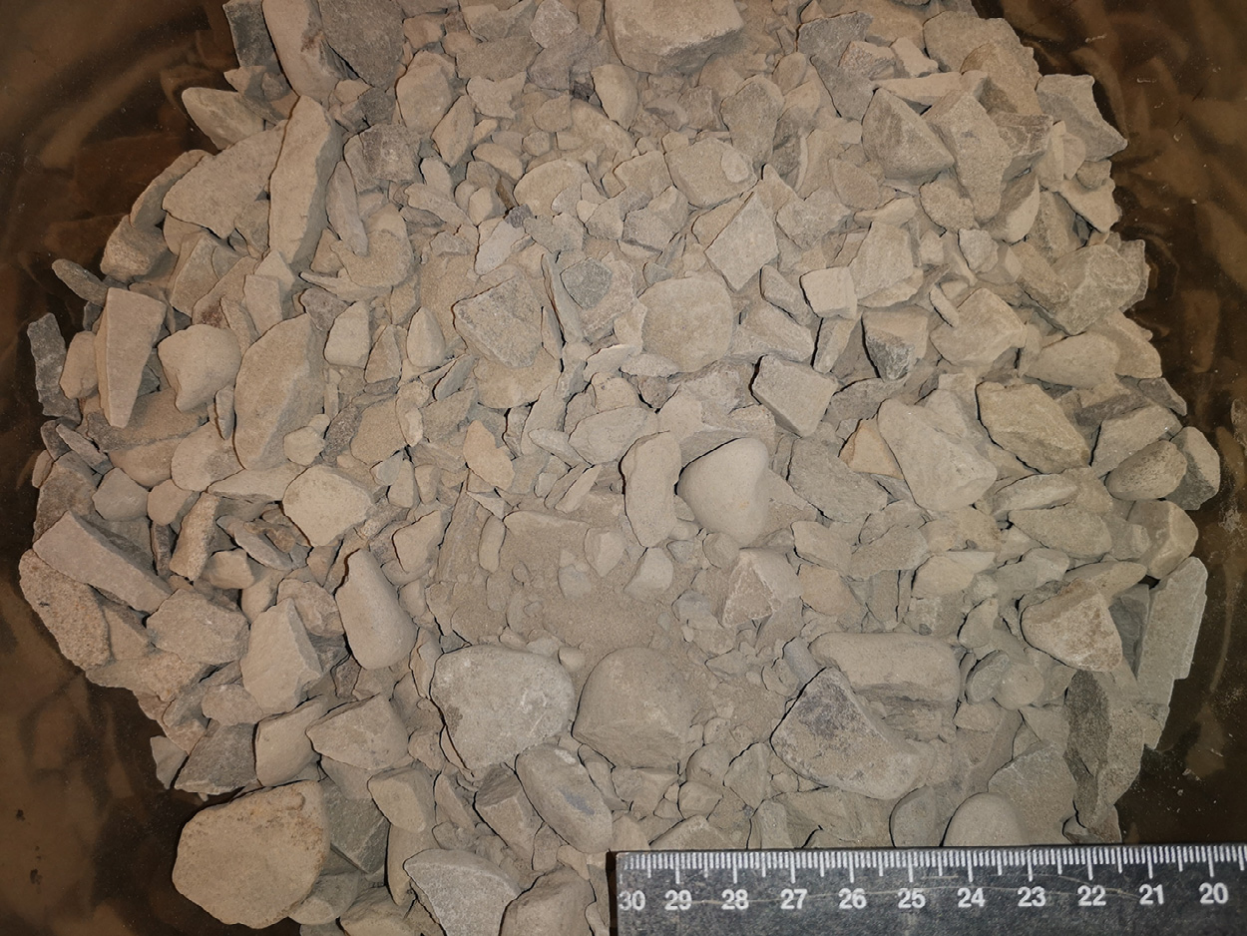
Loose material tested with RLTTs.

A testing campaign was performed in the laboratories of the Department of Civil and Environmental Engineering (Norwegian University of Science and Technology, Trondheim, Norway) to characterize the mechanical properties of the rocks to serve as possible road construction materials. Given the geographical and climatic peculiarity of the Svalbard archipelago, civil structures built in the region must tackle significant engineering challenges [5,6]. Three types of tests were performed: Los Angeles (LA) tests, micro-Deval (MDE) tests and Repeated Load Triaxial Tests (RLTTs). Both LA and MDE tests were accomplished by investigating particle size comprised between 10 and 14 mm as indicated in the corresponding codes [7,8], five parallel samples were evaluated for each test. When it comes to RLTTs, the grading curve corresponding to a typical base layer used in Norwegian roads was tested [1]; the gradation is displayed in Fig. 10 and the loose material is depicted in Fig. 11.

**Fig. 12:**
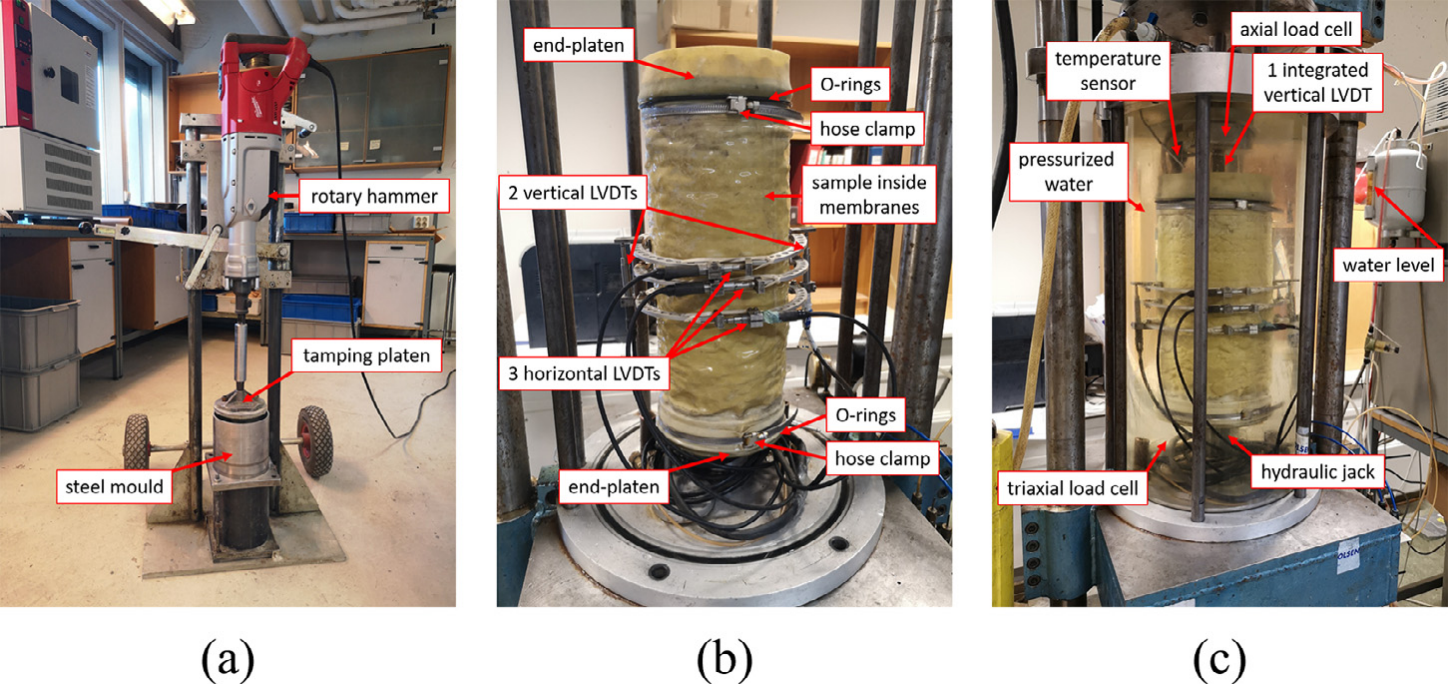
RLTT sample operations: compaction (a), instrumentation (b) and testing (c).

The RLTT thoroughly characterizes the mechanical properties of the rock materials, the two important results that can be evaluated from the test are the resilient modulus *M_R_* and the resistance against permanent deformation [9], [10], [11]. A total of four samples was tested with RLTTs, specimens 1 and 2 had water content *w* = 2% and specimens 3 and 4 had water content *w* = 7% (percentage in mass), each sample had a dry mass of 11,000 g. The preparation of the specimens was accomplished according to a precise order. Initially, the total mass mixed with the desired amount of water was divided in five plastic bags and rest overnight to ensure a uniform water distribution. The material inside each plastic bag corresponded to the grading curve displayed in Fig. 10. Subsequently, the content of the five plastic bags formed the testing sample composed by five layers: each layer was compacted inside a steel mould employing a Milwaukee 2″ SDS Max rotary hammer (hammer weight 12 kg, work per blow 27 N•m, tamping time 25 s) as reported in Fig. 12a.

The dimensions of the created specimen was 150 mm in diameter and 300 mm in height, it was possible to asses bulk and dry density at this point [2]. The sample was extracted from the mould with a customized ejection tool and the specimen was covered by two latex membranes, four O-rings, two hose clamps and two metal end-platens; afterwards, the specimen was placed into the RLTT device and two vertical LVDTs and three radial LVDTs were mounted on the specimen (Fig. 12b).

Subsequently, the RLTT chamber was sealed and filled with water. The RLTT apparatus exerted two types of actions, namely a vertical dynamic pressure (*σ_d_*, deviatoric stress) and a uniform confining pressure (*σ_3_*, triaxial or confining stress). The former one was applied by a hydraulic jack located under the specimen, the latter one was applied by pressurized water. The hydraulic jack integrates an additional third axial LVDT. Fig. 12c displays the operating RLTT device. Table 2 reports the stress paths accomplished for each RLTT according to the Multi-Stage Low Stress Level (MS LSL) [12]: a RLTT is composed of five loading sequences and each sequence, formed by six steps, corresponds to a precise combination of *σ_d_* and *σ_3_*. For each step, *σ_3_* is constant while *σ_d_* is repeated 10 000 times according to a sinusoidal pattern varying from a minimum of 5 kPa (to ensure contact between the top end-platen and the load cell) and the proper maximum value reported in Table 2. A sequence comes to a halt after the completion of the six steps or if the axial permanent deformation measured by the integrated axial LVDT reaches 0.5%.

**Table 2:** Stress path for the Multi-Stage Low Stress Level (MSL SL) RLTT (data in kPa).

	Sequence 1		Sequence 2		Sequence 3		Sequence 4		Sequence 5	
	*σ_t_*	*σ_d_*	*σ_t_*	*σ_d_*	*σ_t_*	*σ_d_*	*σ_t_*	*σ_d_*	*σ_t_*	*σ_d_*
**Step 1**	20	20	45	60	70	80	100	100	150	100
**Step 2**	20	40	45	90	70	120	100	150	150	200
**Step 3**	20	60	45	120	70	160	100	200	150	300
**Step 4**	20	80	45	150	70	200	100	250	150	400
**Step 5**	20	100	45	180	70	240	100	300	150	500
**Step 6**	20	120	45	210	70	280	100	350	150	600

For a constant value of *σ_3_* and a variation in the dynamic deviatoric stress *Δσ_d,dyn_*, the resilient modulus *M_R_* is determined as

(1) $$M_{R}=\frac{\Delta \sigma_{d,dyn}}{\varepsilon_{a,el}},$$

with *ε_a,el_* the average axial resilient strain measured by the three axial LVDTs. among the possible formulations that can be used to efficiently display *M_R_* [13], the Hicks & Monismith model [14] is largely adopted to efficiently interpret the empirical data

(2) $$M_{R}=k_{1}\sigma_{a}{\left(\frac{\theta }{\sigma_{a}}\right)}^{k_{2}},$$

where *σ_a_* is a reference pressure (100 kPa) and *k_1_, k_2_* parameters are evaluated by regression analysis. Several formulations exist to clearly display the development of permanent deformations [15]. Categorising each RLTT load step upon the average strain rate $\overset{\cdot }{\varepsilon }$ for the cycles from 5 000 to 10 000, the Coulomb approach defines the mobilized angle of friction *ρ* and the angle of friction at incremental failure *φ* and thus identifies three different behaviours: elastic ($\overset{\cdot }{\varepsilon }$ < 2.5 10^−8^), elasto-plastic (2.5 • 10^−8^ < $\overset{\cdot }{\varepsilon }$ < 1.0 10^−7^) and failure ($\overset{\cdot }{\varepsilon }$ > 1.0 10^−7^) [16]. The equations for the elastic limit line and incremental failure line are

(3) $$\sigma_{d}=\frac{2\sin\rho \;\left(\sigma_{3}+a\right)}{1-\sin\rho },$$

(4) $$\sigma_{d}=\frac{2\sin\varphi \;\left(\sigma_{3}+a\right)}{1-\sin\varphi },$$

where the apparent attraction *a* is assumed to be 20 kPa [17].

## CRediT Author Statement

**Diego Maria Barbieri:** Conceptualization, Methodology, Formal analysis, Investigation, Data curation, Writing - Original Draft, Visualization. **Jean-Gabriel Dorval:** Conceptualization, Methodology, Investigation, Resources, Data curation, Writing - Original Draft, Visualization. **Baowen Lou:** Methodology, Data curation, Writing - Original Draft, Visualization. **Chen Hao:** Methodology, Data curation, Writing - Original Draft, Visualization. **Benan Shu:** Methodology, Data curation, Writing - Original Draft, Visualization. **Fusong Wang:** Methodology, Data curation, Writing - Original Draft, Visualization. **Inge Hoff:** Conceptualization, Methodology, Investigation, Data curation, Visualization, Supervision, Project administration.

## Declaration of Competing Interest

The project was awarded a research grant from the Nordic Road Association (NVF).

## Appendix Supplementary materials

Image, application 1
