# Supplementary material for: Dataset regarding the mechanical characterization of sedimentary rocks derived from Svalbard for possible use in local road constructions
Source: Data Brief. 2021 Jan 9;34:106735. doi: 10.1016/j.dib.2021.106735 (PMC7815468; doi:10.1016/j.dib.2021.106735)
Supplement: Supplementary file 1 [file mmc1.docx]

**Diego Maria Barbieri**

Conceptualization, Methodology, Formal analysis, Investigation, Data curation, Writing - Original Draft, Visualization

**Jean-Gabriel Dorval**

Conceptualization, Methodology, Investigation, Resources, Data curation, Writing - Original Draft, Visualization

**Baowen Lou**

Methodology, Data curation, Writing - Original Draft, Visualization

**Chen Hao**

Methodology, Data curation, Writing - Original Draft, Visualization

**Benan Shu**

Methodology, Data curation, Writing - Original Draft, Visualization

**Fusong Wang**

Methodology, Data curation, Writing - Original Draft, Visualization

**Inge Hoff**

Conceptualization, Methodology, Investigation, Data curation, Visualization, Supervision, Project administration
